# Contribution of PGAP3 co‐amplified and co‐overexpressed with ERBB2 at 17q12 involved poor prognosis in gastric cancer

**DOI:** 10.1111/jcmm.17828

**Published:** 2023-06-29

**Authors:** Dong Wang, Siyu Hao, Hongjie He, Jian Zhang, Ge You, Xin Wu, Rui Zhang, Xiangning Meng, Xiaobo Cui, Jing Bai, Songbin Fu, Jingcui Yu

**Affiliations:** ^1^ Scientific Research Centre The Second Affiliated Hospital of Harbin Medical University Harbin China; ^2^ Key Laboratory of Preservation of Human Genetic Resources and Disease Control in China (Harbin Medical University) Ministry of Education Harbin China

**Keywords:** co‐amplification, co‐overexpression, ERBB2, gastric cancer, PGAP3

## Abstract

The locus at 17q12 erb‐b2 receptor tyrosine kinase 2 (ERBB2) has been heavily amplificated and overexpressed in gastric cancer (GC), but it remains to be elucidated about the clinical significance of the co‐amplification and co‐overexpression of PGAP3 gene located around ERBB2 in GC. The profile of PGAP3 and ERBB2 in four GC cell lines and tissue microarrays containing 418 primary GC tissues was assessed to investigate the co‐overexpression and clinical significance of the co‐amplified genes, and to evaluate the impact of the co‐amplified genes on the malignancy of GC. Co‐amplification of PGAP3 and ERBB2 accompanied with co‐overexpression was observed in a haploid chromosome 17 of NCI‐N87 cells with double minutes (DMs). PGAP3 and ERBB2 were overexpressed and positively correlated in 418 GC patients. Co‐overexpression of the PGAP3 and ERBB2 was correlated with T stage, TNM stage, tumour size, intestinal histological type and poor survival proportion in 141 GC patients. In vitro, knockdown of the endogenous PGAP3 or ERBB2 decreased cell proliferation and invasion, increased G1 phase accumulation and induced apoptosis in NCI‐N87 cells. Furthermore, combined silencing of PGAP3 and ERBB2 showed an additive effect on resisting proliferation of NCI‐N87 cells compared with targeting ERBB2 or PGAP3 alone. Taken together, the co‐overexpression of PGAP3 and ERBB2 may be crucial due to its significant correlation with clinicopathological factors of GC. Haploid gain of PGAP3 co‐amplified with ERBB2 is sufficient to facilitate the malignancy and progression of GC cells in a synergistic way.

## 1 INTRODUCTION

Genomes instability, including gain at chromosome 17q12, is frequently observed in gastric cancer (GC). Amplified DNA can be organized as chromosomal homogeneously staining region (HSR)^1^ or extrachromosomal double minutes (DMs)^2^ or distributed at various locations in the genome. These amplifications are usually correlated with oncogene overexpression and clinical tumour aggressiveness.^3^ Furthermore, amplicons pinpoint candidate oncogenes as they are a visible representation of the genes whose expression promotes tumour growth.^4^ While some amplicons may harbour known oncogenes ‘driving’ amplification, amplicon rarely comprises single gene in tumour. The potential functional contribution of co‐amplified genes remains largely unexplored.^5^ Studies have found that multiple expressed genes presented usually in a single amplicon may provide a growth advantage to cells.^6^ Therefore, the identification of genes with increased expression in a core amplified region could be essential.

A combined investigation of the genes refer to erb‐b2 receptor tyrosine kinase 2 (ERBB2) could facilitate the identification of a target therapeutic regimen for GC patients.^7^ The locus at 17q12 was well known because it is where ERBB2 gene located, which has been heavily amplificated and overexpressed in tumours (including GC).^8^ PPP1R1B‐ERBB2‐GRB7 locus at 17q12 was frequently amplified in GC,^9^ it is generally considered to be an oncogenomic recombination hotspot around the locus at human chromosome 17q12.^10^ In GC, the frequency of ERBB2 amplification has been reported to be in the range from 7% to 27%, and this amplification has been established to correlate with an intestinal type histology and poor survival.^11^ Studies demonstrated that the rate of ERBB2 overexpression is 6%–30% in GC.^12^ Previous studies indicated that the ERBB2 amplicon included STARD3 and GRB7 genes, and the co‐localization and co‐amplification of these genes with ERBB2 suggested a region of genomic amplification in breast cancer that extends beyond ERBB2.^13^, ^14^ In addition to STARD3 and GRB7, post‐GPI attachment to proteins phospholipase 3 (PGAP3) is also one of the genes which are located near ERBB2 around the locus at 17q12. PGAP3 and ERBB2 were found to be amplified and overexpressed in GC by array‐based comparative genomic hybridization (aCGH) and gene expression microarray analyses.^9^, ^15^ However, the implication of PGAP3 co‐amplified and co‐overexpressed with ERBB2 at 17q12 in GC remains largely unknown.

Our previously unpublished result by aCGH indicated the highest amplification of ERBB2 amplicon on 17q12 in GC cell line NCI‐N87 with DMs. In this study, we investigated the clinical and biological implications of PGAP3 co‐amplification with ERBB2 in gastric tumorigenesis in vitro, and demonstrated that the aberrant expression of these genes is associated with the malignancy of GC cells.

## 2 MATERIALS AND METHODS

### 2.1 Cell lines and tissue chips

The GC cell lines NCI‐N87 and AGS were obtained from American Type Culture Collection (ATCC, USA), BGC‐823 and HGC‐27 were from the Cell Resources Center of Shanghai Life Sciences, Chinese Academy of Sciences. Gastric epithelial cell line GES‐1, which was kindly provided by Laboratory of Medical Genetics (China medical university). All cell lines were cultured in RPMI‐1640 medium (Gibco BRL, 2457402) supplemented with 10% foetal bovine serum (FBS) (Absin, abs972) apart from AGS which was cultured in F‐12k medium (Gibco BRL, 2142045) supplemented with 10% FBS. All cell lines were maintained in 5% CO_2_ at 37°C.

A set of primary GC tissue chips (Serial numbers: HStm‐Ade180Sur‐01, HStm‐Ade180Sur‐02, HStm‐Ade180Sur‐06, HStm‐Ade180Sur‐07 and HStm‐Ade180CS‐01; Shanghai Outdo Biotech Co. Ltd.), were used in the immunohistochemistry (IHC) analysis, which contained GC tissue (For PGAP3: 418 cases; For ERBB2: 418 cases), intestinal metaplasia (IM) tissues (For PGAP3: 29 cases; For ERBB2: 33 cases) and noncancerous gastric (NG) tissues (For PGAP3: 381 cases; For ERBB2: 368 cases), after excluding factors such as exfoliation, too few cancer tissues and tissue duplication. None of the patients has received pre‐operative chemotherapy, radiotherapy or both. GC tissue samples came with detailed clinical information, including gender, age, stage and histological type. There were 343 cases with follow‐up information, 75 cases without follow‐up information and 416 cases fitted the Lauren's classification (Intestinal type: 223 cases; Mixed type: 71 cases; Diffuse type: 122 cases). Therefore, a total of 418 patients were included in the correlation analysis and 343 patients were included in the survival analysis, including 222 death cases and 121 survival cases. All studies involving human samples were carried out in accordance with The Code of the World Medical Association (Declaration of Helsinki). Before the experiment, all the tissue chips were approved by the Ethics Committee of Shanghai Outdo Biotech Co. Ltd. (YB M‐05‐02).

### 2.2 Metaphase chromosome preparation and karyotype analysis

Metaphase spread preparation was done following standard protocol. Briefly, cells were exposed to colchicine at the final concentration of 0.2 μg/mL for 1–1.5 h before harvesting. Supernatant was removed after centrifugation, then treated with 0.075 mol/L preheated KCl solution for about 14–15 min at 37°C, followed by 15 min fixation. After centrifugation, cells were resuspend and dropped onto slides. Before stained with Giemsa, the karyotypes of NCI‐N87 cells were treated with trypsin at 37°C. Images were obtained using a microscope (NIKON YS100), the karyotypes of the cells were determined using standard cytogenetic techniques.

### 2.3 Real‐time PCR

Genomic DNA from GC cell lines was isolated with the QIAamp DNA mini kit (Qiagen, 51306, Germany) according to the manufacturer's protocol. Total RNA from GC cell lines was extracted using TRIzol reagent (Invitrogen, 66003, USA) and reverse transcribed into cDNA using the Transcriptor First Strand cDNA Synthesis Kit (Roche, 04897030001, Germany). The sequences of the PCR primers used in the study are listed in Table S1. All PCR reactions were performed in Roche Light Cycler 480 system (Roche, Switzerland). The relative level of each gene were normalized by β‐actin, and was shown as fold differences (2^−ΔΔCT^).

### 2.4 Western blotting

Cells were retrieved into RIPA buffer containing protease inhibitor. The total protein concentration was determined by a DU800 Protein Analyser (Beckman). Proteins were resolved on SDS‐PAGE and electron transferred to a PVDF membrane. The membranes were first blocked in TBS containing 5% powdered skimmed milk, and was hybridized with the primary antibodies against ERBB2 (Sigma, SAB5700151, 1:1000) and PGAP3 (Abcam, ab129264, 1:500) overnight. Then the blots were incubating with the corresponding second anti‐rabbit antibody or anti‐mouse antibody (Rockland, 1:20000). β‐Actin (ZSGB‐BIO, TA‐09, 1:500) was used as an internal control. The results were visualized using the Odyssey Infrared Imaging System (Li‐COR, USA). For genes silencing efficiency validation, anti‐ERBB2 antibody (Immunoway, YM3045, 1:1000) and anti‐PGAP3 antibody (Immunoway, YN5206, 1:1000) were used as primary antibodies, peroxidase‐conjugated goat anti‐rabbit (ZSGB‐BIO, ZB‐5301, 1:10000) used as second antibody, chemiluminescence detection with ECL substrate (Seven, SW181‐01, China) using the Chemiluminescence Apparatus (CLINX, 6300, China).

### 2.5 Immunohistochemistry

IHC was performed following standard protocol with anti‐PGAP3 (Abcam, ab129264, 1:2000) and anti‐ERBB2 antibody (Abcam, ab8054, 1:75). IHC score was represented by staining intensity score and staining positive rate score. Staining intensity score was classified into five grades from low to high intensity: 0 (negative), 0.5 (0.5+), 1 (1+), 2 (2+), 3 (3+). Staining positive rate score ranged from low to high staining (0%–100%). The total IHC staining score was calculated as the ‘staining intensity score’ multiplied by ‘staining positive rate score’^16^ (0%–300%). Total cytoplasmic/membrane IHC score ≤ 100% was defined as low expression, >100% was defined as high expression.

### 2.6 Transient transfection

3 × 10^3^ cells/well were seeded into 24‐well plate with 500 μL RPMI‐1640, and transfected using lipofectamine RNAiMAX Reagent (Thermo Fisher, 13778030, USA) according to the manufacturer's protocol. SiRNAs for PGAP3 and ERBB2 were purchased from Beijing VIEWSOLID Biotechnology Co., Ltd., and the sequences are listed in Table S2.

### 2.7 Cell cycle and apoptosis assay

Cells were seeded in a six‐well plate with 1 × 10^4^ cells/well, followed by transfected with lipofectamine RNAiMAX transfection reagent. Three groups were set up in total. Negative control (NC) group was transfected with si‐NC, siPGAP3 group was transfected with siPGAP3‐189, and siERBB2 group was transfected with siERBB2‐2. Cells were digested 48 h after transfection, PI staining or Annexin V and PI double‐staining were performed according to Annexin‐V‐FLUOS Staining Kit (Roche, 11988549001, Germany), and the percentage of cells was analysed by flow cytometry.

### 2.8 Cell proliferation assay, colony formation and invasion assay

Cells proliferation were measured using CCK‐8 solution (Dojindo, CK04, Japan) for 7 days over time. Cells were seeded into a 96‐well cell culture plate with 3 × 10^3^ cells/well. The dosage of Lipofectamine RNAiMAX was 0.3 μL/well, and the dosage of siRNA was 3 pmol/well. The medium was removed 72 h after transfection, and cells were cultured with RPMI‐1640 medium containing 10% CCK‐8 kits for 2 h. The absorbance value was obtained at 450 nm by the microplate tester.

In colony formation assay, 1 × 10^4^ cells were seeded into a six‐well plate after transfection. After 2 weeks, the number of cell colonies was determined by fixed staining with Giemsa to measure the ability of cells to proliferate.

In cell invasion assay, cells in each group were transfected with siRNA (siPGAP3‐189, siERBB2‐2), 4 × 10^3^ cells/well were seeded into the upper layer of Transwell chamber (BD Biosciences, 353097, USA) precoated with 100 μL Matrigel. 600 μL medium containing 10% FBS was added to the lower layer of the chamber. After 48 h, the cells in the lower membrane were fixed in 4% paraformaldehyde, stained with DAPI and photographed under a fluorescence microscope. The invasion ratio was calculated as the percentage of invasive cells across the chamber to the total number of seeded cells.

### 2.9 Data collection and analysis

The expression levels of PGAP3 and ERBB2 were obtained from the Cancer Cell Line Encyclopedia (CCLE) database, which contains data on 45 cell lines involving GC. This information was accessed through the website https://sites.broadinstitute.org/ccle. The RNA levels of genes in 408 GC tissues were analysed using Pearson correlation analysis, along with 211 normal gastric tissues (36 normal and 175 stomach) through the Gene Expression Profiling Interactive Analysis (GEPIA) databases available at http://gepia2.cancer‐pku.cn.

### 2.10 Statistical analysis

Data are shown as mean ± SEM. Statistical analysis was performed using SPSS.19 software. GraphPad Prism 8 was used for figure drawing. *t*‐Tests were used to compare the difference of expression and amplification for PGAP3 and ERBB2 in tumour cells. Kruskal–Wallis test was used to analyse the expression differences of PGAP3 and ERBB2 in GC, IM and NG tissues. Bivariate correlation analysis was performed by Pearson test, chi‐squared test was used to analyse the correlation between the expression of PGAP3 or ERBB2 and clinical indicators. Kaplan–Meier analysis was used for survival curves with log‐rank tests for survival comparisons between groups. A univariate cox proportional hazards model was applied to examine the association between overall survival and overexpression of PGAP3 and ERBB2 as well as other clinical parameters. Stepwise multivariate survival analysis was carried out using cox proportional hazards model. Statistical significance was determined by *p* < 0.05.

## 3 RESULTS

### 3.1 Co‐amplification and co‐overexpression of PGAP3 and ERBB2 in GC cell line NCI‐N87

**FIGURE 1:**
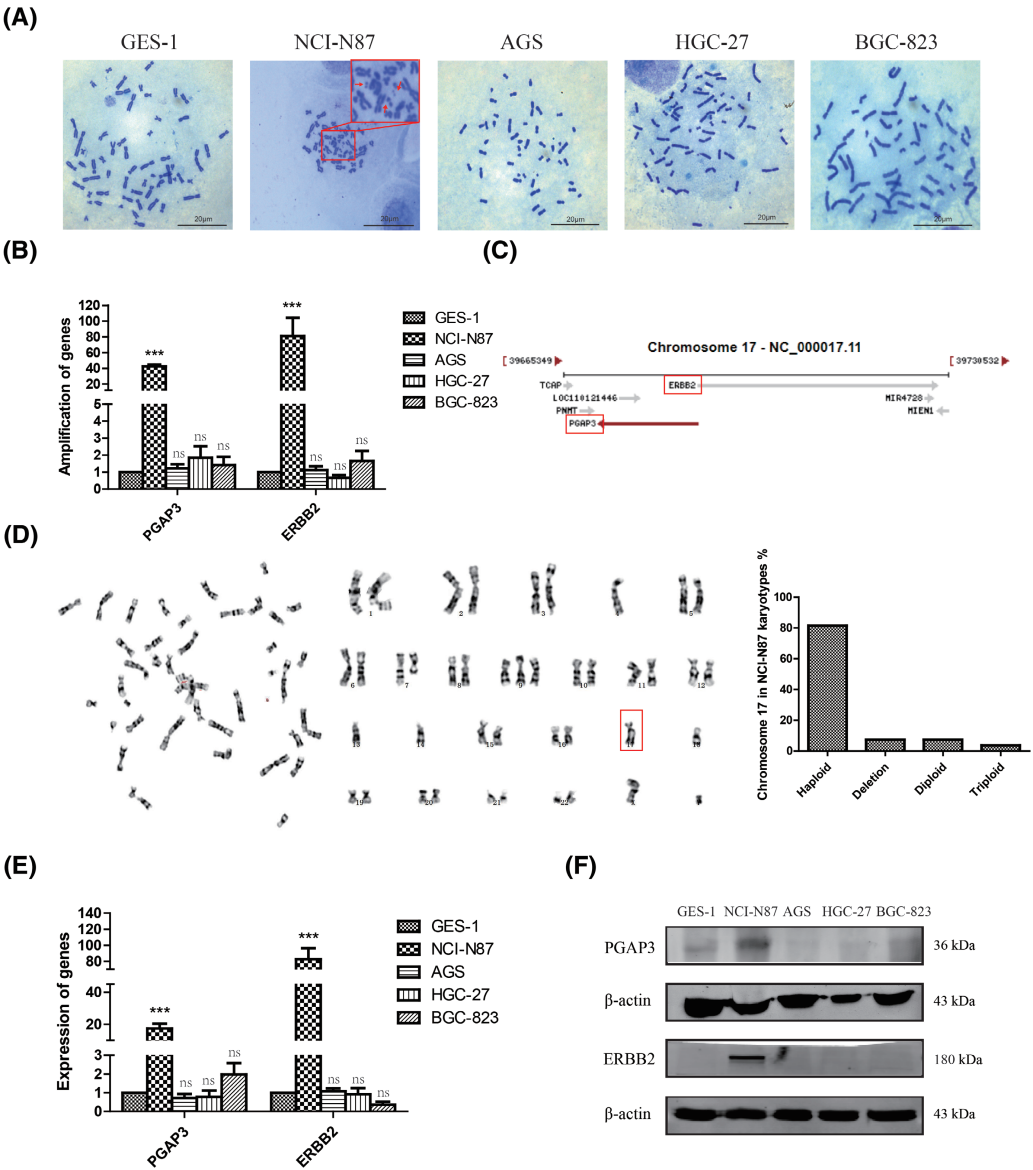
Haploid gain and co‐overexpression of PGAP3 and ERBB2 in GC cell lines. (A) Metaphase chromosome karyotypes from gastric epithelial cell line GES‐1 and GC cell lines (NCI‐N87, AGS, HGC‐27, BGC‐823). Representative DMs are marked by arrowheads in NCI‐N87 cells (magnification, ×1000). (B) The amplification level of PGAP3 and ERBB2 by real‐time PCR in GC cell lines. (C) The genomic location of PGAP3 and ERBB2 on chromosome 17. (D) The chromosome karyotype (left) and the proportion of haploid karyotype (right) in NCI‐N87 cells. (E) The mRNA level of PGAP3 and ERBB2 in GC cell lines. (F) The protein level of PGAP3 and ERBB2 in GC cell lines. (ns, no significance; ***p < 0.001).

As amplified genes are typically found on chromosomal as the form of HSR or DM, the karyotype of GC cell lines NCI‐N87, AGS, HGC‐27 and BGC‐823 was determined to search for either of these cytogenetic structures. It is visible that NCI‐N87 cells contained numerous number of DMs, while other GC cell lines did not (Figure 1A). Subsequently, the DNA levels of PGAP3 and ERBB2 were detected in NCI‐N87 (DM^+^ cell line) and AGS, HGC‐27 and BGC‐823 (DM^−^ cell line) by Real‐time PCR. Compared with control cells (GES‐1), the relative copy number of PGAP3 and ERBB2 genes were significantly increased in NCI‐N87 cells (Figure 1B, ***p < 0.001), and no significant changes in AGS, HGC‐27 and BGC‐823 cells. Co‐amplification of PGAP3 and ERBB2 was consistent with their genomic location on chromosome 17 (https://www.ncbi.nlm.nih.gov/gene/2064) (Figure 1C). Notably, the karyotype of NCI‐N87 cells showed a haploid chromosome 17. In a total of 27 karyotypes of NCI‐N87 cells analysed, the haploid karyotype of chromosome 17 were observed in 81.5% of the total karyotypes (Figure 1D), including 22 haploid of chromosome 17, 2 deletions of chromosome 17, 2 diploids of chromosome 17 and 1 triploid of chromosome 17. This suggests that haploid gain of PGAP3 and ERBB2 on chromosome 17 occurred in majority of NCI‐N87 cells.

Then, the mRNA levels of PGAP3 and ERBB2 were measured by Real‐time PCR. Compared with GES‐1 cells, the transcripts of PGAP3 and ERBB2 were no significant changed in DM^−^ cells, but were markedly increased in NCI‐N87 (DM^+^) cells (Figure 1E, ^***^ *p <* 0.001), accompanied by increased protein levels by western blot (Figure 1F). In addition, we found that the expression of PGAP3 and ERBB2 was the highest in intestinal (tubular) cell lines (Figure S1) in CCLE databases, and their expression was positively correlated in all GC cell lines (Figure S2). Besides, the expression of PGAP3 and ERBB2 were mainly found in metastatic GC cell lines (62.22%) (Figure S3), and after ERBB2 knockdown, PGAP3 decreases in intestinal (tubular) GC cell lines (Figure S4). The co‐amplification and co‐overexpression of PGAP3 and ERBB2 implicated their potential role in gastric tumour pathogenesis.

### 3.2 Co‐overexpression of PGAP3 and ERBB2 in primary GCs

**FIGURE 2:**
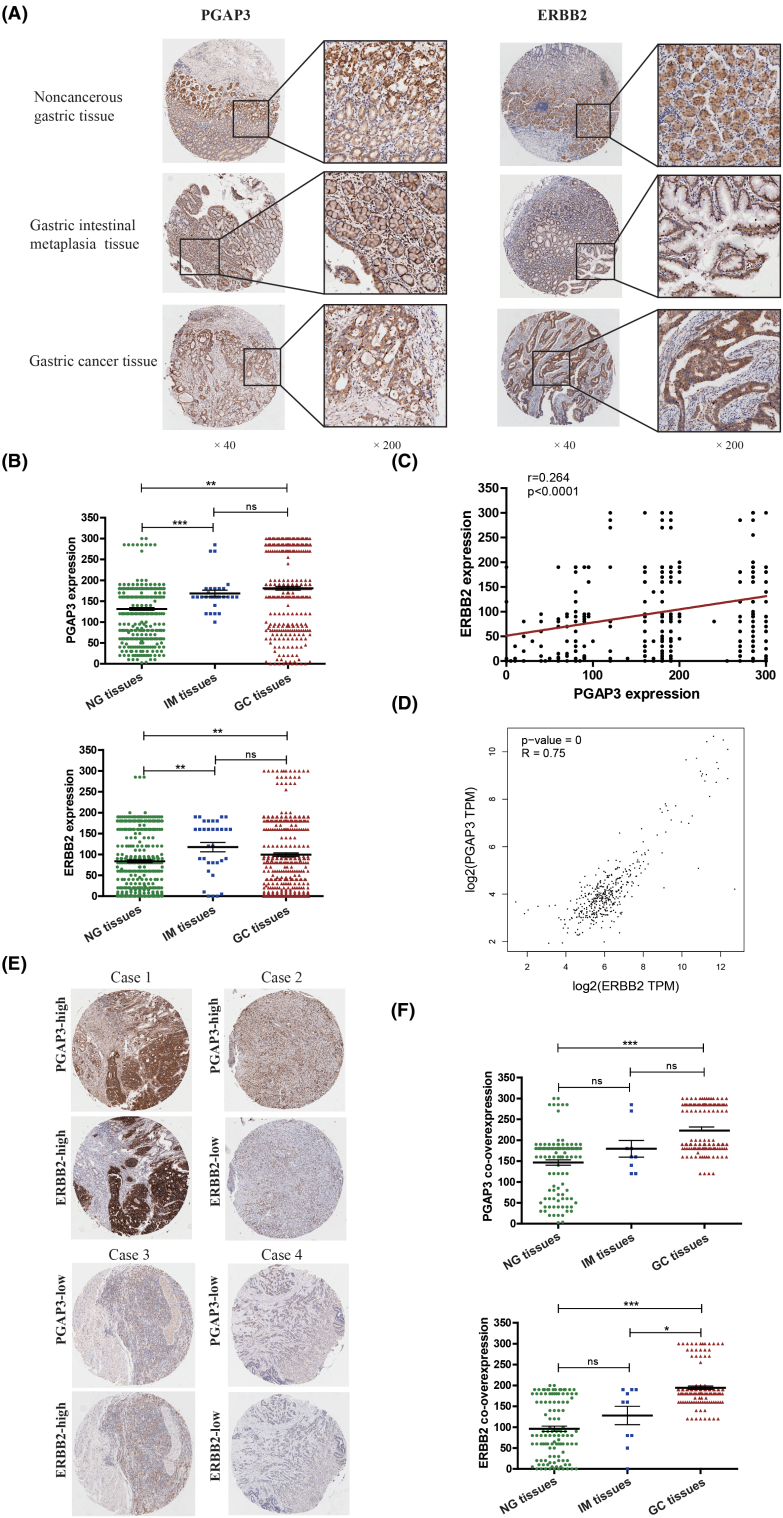
Co‐overexpression of PGAP3 and ERBB2 by IHC in primary GCs. (A) Representative immunoreactivity of PGAP3 and ERBB2 in NG, IM and GC tissues (magnification, ×40 and ×200). (B) Expression of PGAP3 and ERBB2 proteins in NG, IM and GC tissues. (C) The Pearson correlation analysis between the immunoreactivity of PGAP3 and ERBB2 proteins in GC tissues. (D) The Pearson correlation analysis between the RNA levels of PGAP3 and ERBB2 in GC tissues based on GEPIA database. (E) Representative figures of PGAP3 and ERBB2 co‐expression pattern in the same case of GC by IHC (magnification, × 40). (F) Co‐overexpression of PGAP3 and ERBB2 proteins in GC, matched IM and NG tissues. (ns, no significance; *p < 0.05, **p < 0.01, ***p < 0.001).

To assess the significance of PGAP3‐ERBB2 co‐amplification in primary GC, the expression of these genes were analysed by IHC in tissue microarrays (Figure 2A). Results indicated that PGAP3 and ERBB2 showed stronger cytoplasm/membrane staining in GC tissues (IHC score: 180.67 ± 4.038 and 99.49 ± 4.074), IM tissues (IHC score: 168.4 ± 7.937 and 117.6 ± 11.26) and NG tissues (IHC score: 131.54 ± 3.310 and 83.56 ± 3.628) (Table 1). The expression of PGAP3 and ERBB2 in GC tissues and IM tissues were significantly higher than that in NG tissues (***p < 0.0001 and **p = 0.0036; ***p = 0.0001 and **p = 0.0066) (Table 1; Figure 2B). Pearson test revealed a significant positive correlation of expression between PGAP3 and ERBB2 (*r* = 0.264, ***p < 0.001) (Figure 2C), which was consistent with the data of RNA sequencing (*r* = 0.75, ***p < 0.001) from GEPIA Database (Figure 2D).

**TABLE 1:** Expression of PGAP3 and ERBB2 in NG, IM and GC tissues.

	Variables	NG tissue	IM tissue	GC tissue	*p* value
PGAP3	Mean ± SEM	131.54 ± 3.310	168.4 ± 7.937	180.67 ± 4.038	***p < 0.0001
	Number	381	29	418	
ERBB2	Mean ± SEM	83.56 ± 3.628	117.6 ± 11.264	99.49 ± 4.074	**p = 0.0053
	Number	368	33	418	

To further clarify the effect of co‐overexpression of the PGAP3 and ERBB2 in GC patients, we assigned the GC patients into four groups according to gene expression in GC tissues as follows: PGAP3‐low/ERBB2‐low, PGAP3‐low/ERBB2‐high, PGAP3‐high/ERBB2‐low and PGAP3‐high/ERBB2‐high (Figure 2E). PGAP3‐high/ERBB2‐high group means the co‐overexpression of the PGAP3 and ERBB2 in GC tissues. Co‐overexpression for PGAP3 and ERBB2 was found in 141 GC patients, in matched NG tissues (121 and 116) and IM tissues (9 and 10), respectively. Immunoreactivity of PGAP3 and ERBB2 showed stronger cytoplasm/membrane staining of the proteins in GC tissues (IHC score: 223.01 ± 4.607 and 194.50 ± 3.921), IM tissues (IHC score: 168.4 ± 7.937 and 117.6 ± 11.264) and NG tissues (IHC score: 146.67 ± 6.38 and 96.08 ± 6.357). The expression of PGAP3 and ERBB2 were gradually increased in NG tissues, IM tissues and GC tissues (Table 2), with the expression of PGAP3 and ERBB2 in GC tissues significantly higher than that in NG tissues (***p < 0.0001 and ***p < 0.0001) and IM tissues (ERBB2, *p = 0.0154) (Figure 2F) in a gradual way (Table 2).

**TABLE 2:** Co‐overexpression of PGAP3 and ERBB2 in GC, matched IM and NG tissues.

	Variables	GC tissue	IM tissue	NG tissue	*p* value
PGAP3	Mean ± SEM	223.01 ± 4.607	179.44 ± 19.97	146.67 ± 6.38	***p < 0.0001
	Number	141	9	121	
ERBB2	Mean ± SEM	194.50 ± 3.921	128 ± 21.95	96.08 ± 6.357	***p < 0.0001
	Number	141	10	116	

### 3.3 Co‐overexpression of ERBB2 and PGAP3 predicts poor survival in primary GCs

**FIGURE 3:**
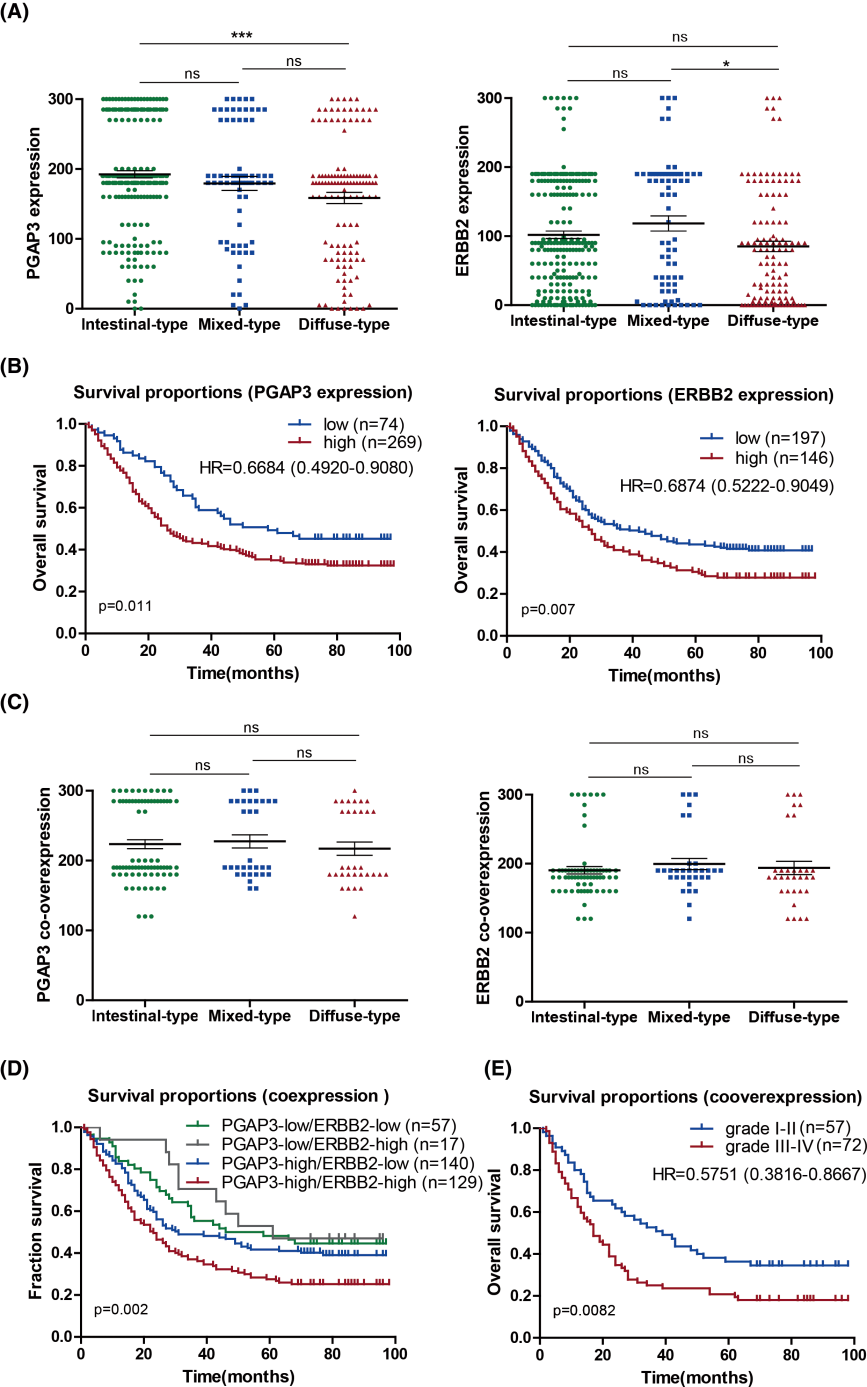
The clinicopathological features and survival analysis of PGAP3 and ERBB2 in GC patients. (A) Expression of PGAP3 or ERBB2 in various types of GC according Lauren type. (B) Survival analysis of PGAP3 and ERBB2 in GC. (C) Co‐overexpression of PGAP3 or ERBB2 in various types of GC according to Lauren classification. (D) Survival analysis of PGAP3‐low/ERBB2‐low, PGAP3‐low/ERBB2‐high, PGAP3‐high/ERBB2‐low and PGAP3‐high/ERBB2‐high groups in GC. (E) Survival analysis of Grade I–II and III–IV with PGAP3‐high/ERBB2‐high group in GC. (ns, no significance; *p < 0.05, ***p < 0.001).

In all 418 GC tissues, the high expression rate for PGAP3 and ERBB2 was found in 322 (77.03%) and 160 (38.28%) GC tissues, respectively. Furthermore, the correlation analysis between gene expression and clinicopathological features indicated that the expression of PGAP3 was significantly correlated with Lauren type (Table S3, *p = 0.013), and the expression of ERBB2 was significantly correlated with T stage (*p = 0.015), tumour size (*p = 0.047) and Lauren type (*p = 0.016) (Table S4). Subsequently, both univariate and multivariate analyses indicated that PGAP3 and ERBB2 were independent prognostic factors for patients with GC (PGAP3: *p = 0.013 and *p = 0.042; ERBB2: **p = 0.008 and *p = 0.033). Besides, tumour size, TNM stage, N metastasis and M metastasis were also significantly correlated with the prognosis of GC patients, as an independent factor of GC patients (Tables S5 and S6). The expression of PGAP3 and ERBB2 was more frequently found in the intestinal histological type, and the expression of PGAP3 in intestinal type of GC was significantly higher than that in diffuse type of GC (***p < 0.001) (Figure 3A). Kaplan–Meier survival analysis in 343 GC patients unravelled that the proportion of total survival is 35%, and overexpression of PGAP3 or ERBB2 has significant poor survival proportion compared with low expression in GC patients, respectively (32.3% and 44.6%, *p = 0.011; 27.4% and 40.6%, **p = 0.007) (Tables S7 and S8, Figure 3B).

Co‐overexpression for PGAP3 and ERBB2 was found in 141 GCs, accounting for 88.13% of ERBB2 high‐expressed GCs. As expected, co‐overexpression of PGAP3 and ERBB2 was also correlated with T stage (*p = 0.01), TNM stage (*p = 0.032), tumour size (*p = 0.032) and Lauren classification (**p = 0.008) (Table 3). Moreover, both univariate and multivariate analyses indicated that co‐overexpression of the PGAP3 and ERBB2 (**p = 0.004 and *p = 0.046), and T stage (***p < 0.001 and *p = 0.048) were significantly correlated with the prognosis of GC patients, as the independent factors of GC patients (Table 4). The intestinal histological type was also more frequently found to carry co‐overexpression (PGAP3‐high/ERBB2‐high) (Figure 3C). Survival analysis indicated that co‐overexpression group showed the worst prognosis among four groups (24.8%) (Figure 3D, **p = 0.002), especially in Grade III–IV GC (17%) (Figure 3E, **p = 0.008), with the survival proportion significantly lower than that in the total survival proportion (24.8% vs. 35%) and (17% vs. 35%) (Table 5). Suggested a synergistic effect of PGAP3 and ERBB2 on the progression and prognosis in GC.

**TABLE 3:** Correlation between PGAP3 and ERBB2 co‐expression and clinicopathological characteristics in GC patients.

Variables	PGAP3 and ERBB2 expression		Total	*χ* ^2^	*p* value
	PGAP3‐low/ERBB2‐low	PGAP3‐high/ERBB2‐high			
Age (years)				0.722	0.395
≤63	38	62	100		
>63	38	79	117		
Sex				0.320	0.571
Female	23	48	71		
Male	53	93	146		
Grade				0.005	0.941
I/II	18	33	51		
III/IV	58	109	167		
T stage				6.634	0.01*
T1/T2	18	15	33		
T3/T4	58	127	185		
N stage				0.281	0.596
N0	19	31	50		
N1/N2/N3	57	111	168		
M stage				0.010	0.922
M0	73	136	209		
M1	3	6	9		
TNM stage				4.609	0.032*
I/II	37	48	85		
III/IV	39	94	133		
Tumour size				4.617	0.032*
≤5 cm	45	61	106		
>5 cm	31	78	109		
Lauren type				9.562	0.008**
Intestinal‐type	30	77	107		
Mixed‐type	13	32	45		
Diffuse‐type	33	33	66		

* *p* < 0.05

** *p* < 0.01.

**TABLE 4:** Univariate and multivariate analyses in GC patients.

Variables	Univariate analysis				Multivariate analysis			
	*p* value	HR	95%CI		*p* value	HR	95%CI	
			Inferior limit	Upper limit			Inferior limit	Upper limit
PGAP3‐low/ERBB2‐low vs. PGAP3‐high/ERBB2‐high	0.004**	1.215	1.063	1.389	0.046*	1.149	1.003	1.316
Age (≤63 vs. > 63)	0.816	1.042	0.737	1.474				
Sex (female vs. male)	0.274	0.818	0.57	1.172				
Grade stage (I/II vs. III/IV)	0.003**	2.038	1.275	3.257	0.058	1.583	0.985	2.545
TNM stage (I/II vs. III/IV)	<0.001	3.329	2.23	4.97	0.057	1.678	0.984	2.861
T stage (T1/T2 vs. T3/T4)	<0.001	4.41	2.054	9.469	0.048*	2.27	1.009	5.111
N stage (N0 vs. N1/N2/N3)	<0.001	3.685	2.175	6.244	0.052	1.973	0.995	3.912
M stage (M0 vs. M1)	0.015*	3.081	1.245	7.62	0.109	2.109	0.846	5.26

* *p* < 0.05

** *p* < 0.01.

**TABLE 5:** Survival proportion of PGAP3 and ERBB2 co‐expression in GC patients.

Group	Number	Death	Survival	Survival rate (%)
PGAP3‐low/ERBB2‐low	57	32	25	43.90
PGAP3‐low/ERBB2‐high	17	9	8	47.10
PGAP3‐high/ERBB2‐low	140	85	55	39.30
PGAP3‐high/ERBB2‐high	129	97	32	24.80
Total number	343	223	120	35.00

### 3.4 Phenotypic plasticity of PGAP3 and ERBB2 knockdown by siRNA in NCI‐N87 cells

**FIGURE 4:**
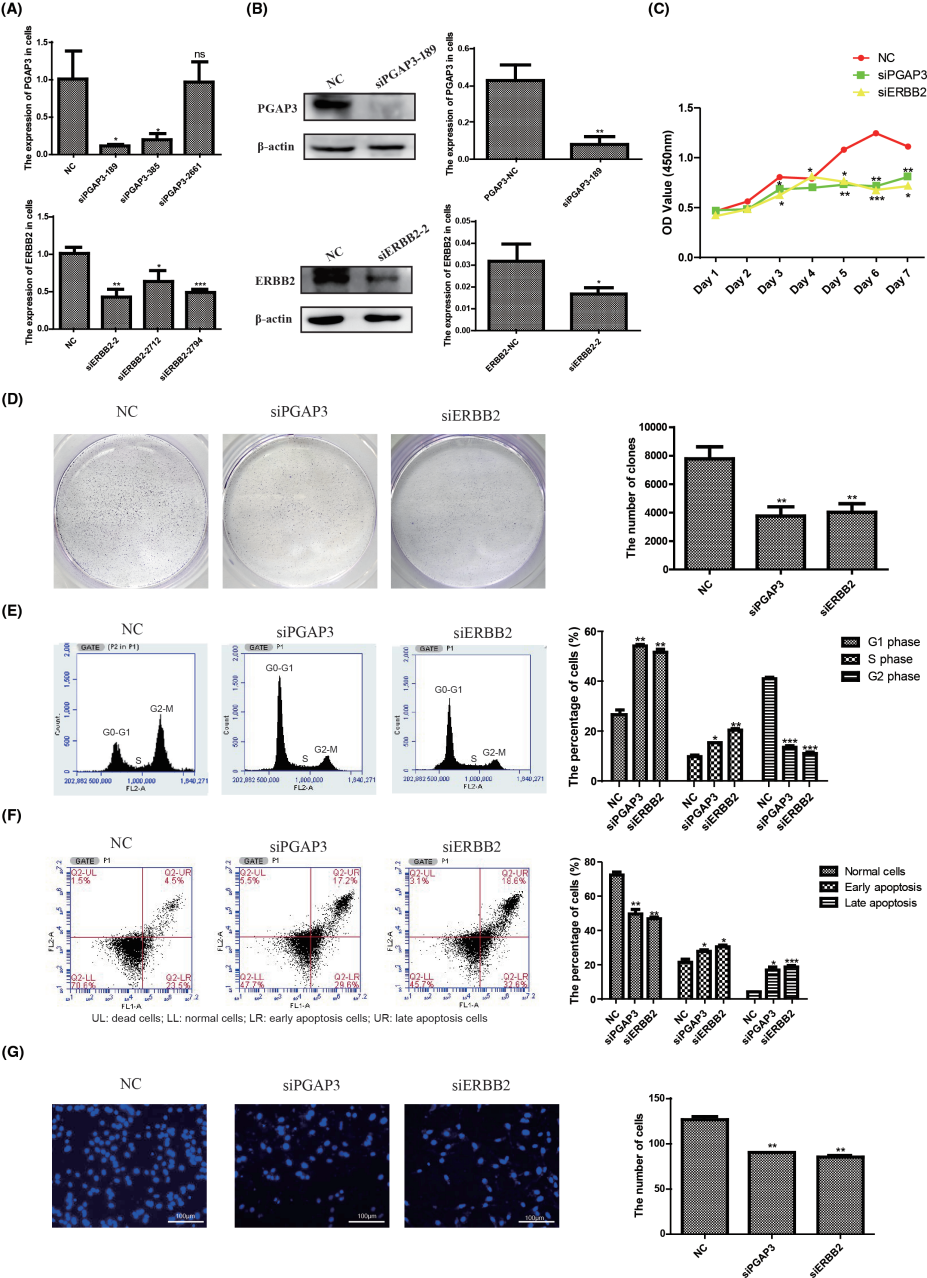
Effects of siRNA‐mediated knockdown of PGAP3 and ERBB2 in NCI‐N87 cells. (A, B) Validation of siRNA knockdown of PGAP3 or ERBB2 by Real‐time PCR and Western blot, respectively. (C, D) Silencing of PGAP3 or ERBB2 led to significant decrease in cell viability. (E, F) Inhibition of PGAP3 or ERBB2 caused cell cycle arrest at G1 and increased apoptosis measured by flow cytometry. (G) Representative images of invading cells through the Matrigel‐coated membrane. The invaded cells were quantified as a percentage of the original seeded cells. Magnification, ×200. (compared to NC, ns, no significance; *p < 0.05, **p < 0.01, ***p < 0.001).

To determine whether the PGAP3 co‐amplified with ERBB2 is a contributing factor to oncogenesis. SiRNA‐mediated knockdown of PGAP3 and ERBB2 was performed to assess the contribution of amplified genes for GC phenotypes. The knockdown efficiency was validated by Real‐time PCR (Figure 4A) and Western blot (Figure 4B), in comparison to transfection of a control vector.

The effect of PGAP3 expression was first examined on cell proliferation by CCK‐8 and clone formation assays. Results showed that knockdown of PGAP3 resulted in decreased cell proliferation, similar to ERBB2 knockdown which led to significant inhibition of cell proliferation in NCI‐N87 cells (Figure 4C,D), suggesting the importance of PGAP3 in gastric carcinogenesis.

The reduced cell proliferation following knockdown of PGAP3 or ERBB2 might result from decreasing cell‐cycle progression or increasing cell death (apoptosis) or both. To further elucidate the mechanism of proliferation alteration, cell‐cycle distribution and apoptosis of GC cells were analysed by Flow cytometry. Knockdown of PGAP3 or ERBB2 in NCI‐N87 cells resulted in G1 accumulation (Figure 4E) and increasing apoptosis cells (Figure 4F), indicating that reduced cell proliferation was attributable to decreased cell‐cycle progression and increased cell apoptosis.

To definite whether PGAP3 expression is required for tumour cell metastasis in NCI‐N87 cells. Boyden chamber assay revealed that the tumour cells transfected with siPGAP3‐189 resulted in a greater decrease of invasion through the Matrigel, compared with the normal cells (NC), indicating that PGAP3 depletion decreased the invasive capabilities of GC cells. The similar result was also observed in the NCI‐N87 cells transfected with siERBB2‐2 (Figure 4G). These data suggest that PGAP3 might be one of the driver genes within the 17q12 amplicon, and may constitute a therapeutic target for GC patients harbouring this amplification.

### 3.5 Assessing additive effects by targeting both PGAP3 and ERBB2

**FIGURE 5:**
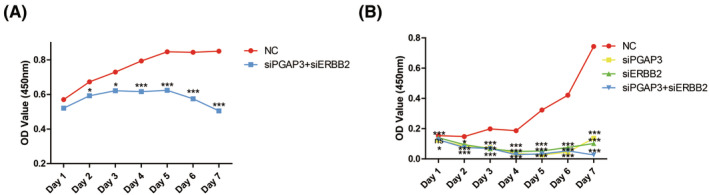
Effects of siRNA‐mediated simultaneous knockdown of PGAP3 and ERBB2 on proliferation in NCI‐N87 cells. (A) Simultaneous silencing of PGAP3 and ERBB2. (B) Silencing of PGAP3 and ERBB2 simultaneously and respectively (compared to NC, ns, no significance; *p < 0.05; ***p < 0.001).

To further investigate whether PGAP3 and ERBB2 might play an additive role. NCI‐N87 cells were co‐transfected with siRNAs targeting PGAP3 and ERBB2. Compared to NC, simultaneous silencing of PGAP3 and ERBB2 led to a clear additive inhibition of NCI‐N87 cell proliferation (Figure 5A). And compared to targeting PGAP3 or ERBB2 alone, simultaneous silencing of PGAP3 and ERBB2 begun to led a clear additive inhibition of cell proliferation in 6–7 days (Figure 5B).

## 4 DISCUSSION

Amplification and/or deletion at chromosome 17 are frequently encountered in GC. In previously studies, we revealed the candidate tumour suppressor genes involved in GC pathogenesis by loss of heterozygosity (LOH) on chromosome 17 in primary GCs.^17^, ^18^ That our previous data of aCGH indicated the highest amplification around ERBB2 at chromosome 17q12 in GC cell line NCI‐N87, has switched our focus to the amplification genes at 17q12 in GC. Although PPP1R1B‐STARD3‐TCAP‐PNMT‐PGAP3‐ERBB2‐MIEN1‐GRB7 located at chromosome 17q12 is frequently amplified in GC,^19^ the cytogenetic characterization and clinical significance of PGAP3 co‐amplified with ERBB2 on the gene loci remain unclear.

Gene amplification represents one of the molecular mechanisms of oncogene overexpression in the tumour.^20^ There has been much evidence that amplified genes in cancer cells usually reside on chromosomal HSR or extrachromosomal DM.^21^ The co‐overexpression and co‐amplification of PGAP3 and ERBB2 in NCI‐N87 cells suggests that the amplicon might be located in DMs in NCI‐N87 cells, which carries DMs.^22^ Notably, we observed the haploid gain of PGAP3 and ERBB2 in chromosome 17 in NCI‐N87 cells, that is, there were more copies of the genes in this haploid chromosome. Previous studies have found that a number of subcultures of colon carcinoma cell line COLO320 lost DMs and gained HSRs after 1–1.5 years.^23^ In present study, we also observed that DMs present only in a few of cells instead of DM in 64% of NCI‐N87 cells described by Park et al.^22^ DM and HSR are two faces of the same coin. It is known that cells harbouring an amplified gene on DM can be replaced by cells with amplified copies integrated as an HSR during propagation under selective pressure.^21^, ^24^ Conversely, activation of fragile sites by aphidicolin or hypoxia in HSR‐containing cells also generates DMs.^25^ Furthermore, studies exhibiting signature of the intestinal type of GC in NCI‐N87 cells.^26^ Our finding suggested that co‐amplification of PGAP3 and ERBB2 as HSR in NCI‐N87 cells may have biological significance for intestinal type GC development.

Recent studies indicated that PGAP3 might be a potentially oncogene, overexpression of which was an independent survival predictors in gastric and distal oesophageal adenocarcinomas.^27^, ^28^ Interestingly, we find that the IHC score of co‐overexpressed PGAP3 and ERBB2 were gradually increased in NG tissues, IM tissues and GC tissues, which indicated the involvement of PGAP3 in the progression from benign to malignant transformation. IM is a part of stepwise sequence of alterations of the gastric mucosa and one of the main premalignant lesions of intestinal‐type GC,^29^ researchers have found that de novo expression of CDX1, CDX2 and MUC2 were highly correlated with IM development.^30^ In our study, the expression of PGAP3 was positively correlated with expression of CDX1 (*R* = 0.2, **p = 0.0028), CDX2 (*R* = 0.23, ***p = 0.00069) and MUC2 (*R* = 0.18, **p = 0.0082) in gastric tissues, but not in GC tissues (CDX1: *R* = 0.029, *p* = 0.56; CDX2: *R* = 0.015, *p* = 0.77; MUC2: *R* = −0.027, *p* = 0.59) (Figure S5) based on GEPIA Database. These results strongly implicate PGAP3 may act as a new early predicting marker in premalignant GC.

In our study, co‐overexpression of PGAP3 and ERBB2 was found in 88.13% ERBB2 high expressed GC patients and the expression of two genes were positively correlated, suggesting that PGAP3‐ERBB2 co‐expression may be common in GC. Moreover, we also found that co‐overexpression of the PGAP3 and ERBB2 was correlated with T stage, TNM stage, tumour size and intestinal tumour phenotype in GC. Studies have already reported that ERBB2 amplification is an exclusive event of intestinal type GC.^31^, ^32^ Our data corroborated previous findings, further suggesting that the co‐amplification of PGAP3 and ERBB2 might be fundamental in the oncogenesis process.

Overexpression of PGAP3 or ERBB2 has significant poor survival proportion compared with low expression in GC patient, respectively (32.3% vs. 27.4%). Importantly, survival analysis showed that co‐overexpression group (PGAP3‐high/ERBB2‐high) contributed to the worst prognosis in patients with GC (24.8%), indicated an important and synergistic effect of PGAP3 and ERBB2 on the prognosis of GC. This suggests that the combination anti‐PGAP3 and anti‐ERBB2 agents may augment the efficacy of ERBB2‐targeted therapy, making it a better treatment strategy for stage III/IV GC.^33^ Alternatively, a novel molecular subtype could be established based on distinct tissue gene expression patterns involving PGAP3 and ERBB2 in GC, which could be novel target for personalized treatment.^34^ Additionally, PGAP3 silencing alone has reversed the malignant phenotype of NCI‐N87 cells in vitro, implying that PGAP3, not just a cooperator with ERBB2, may also play an important role in the progression of GC.

Co‐amplification caused co‐overexpression of PGAP3 and ERBB2 may have functional implication in GC pathogenesis. Our in vitro assays indicated that targeting PGAP3 or ERBB2 alone could significantly reduce cell proliferation and metastasis. Whether targeting combinations of PGAP3 and ERBB2 might provide additive effects? By targeting PGAP3 together with ERBB2, cell proliferation was significantly abrogated in an additive effect, compared to targeting PGAP3 or ERBB2 alone, suggesting that expression of co‐amplified genes is needed to sustain the growth of GC cells. Our data also first discovered that gain of the gene copies at 17q12 by chromosomal segments amplification event in haploid chromosome might be associated with GC progression, which was previously speculated.^9^, ^15^

## 5 CONCLUSION

On the whole, the enhanced co‐expression of ERBB2 and PGAP3 in NCI‐N87 cells implied their important function in GC cells. Furthermore, the specific co‐overexpression of ERBB2 and PGAP3 in intestinal type in primary GCs suggested that the co‐overexpression of the co‐amplified genes has a closely relationship with the clinical pathological types. Our finding underlines that therapy targeting PGAP3‐ERBB2 amplicon may provide additional benefit over targeting ERBB2 alone for patients with tumours carrying 17q12 amplification. Further study is necessary to determine whether GC cells may become addicted to the amplification of several genes that reside in the ERBB2 amplicon, especially in vivo.

## AUTHOR CONTRIBUTIONS

**Dong Wang:** Investigation (equal); methodology (equal); software (equal); writing – original draft (equal). **Siyu Hao:** Investigation (equal); methodology (equal); software (equal); writing – original draft (equal). **Hongjie He:** Software (equal); visualization (equal). **Jian Zhang:** Software (equal); visualization (equal). **Ge You:** Software (equal); validation (equal). **Xin Wu:** Software (equal); visualization (equal). **Rui Zhang:** Software (equal); visualization (equal). **Xiangning Meng:** Investigation (equal); validation (equal). **Xiaobo Cui:** Investigation (equal); validation (equal). **Jing Bai:** Investigation (equal); validation (equal). **Songbin Fu:** Investigation (equal); writing – review and editing (equal). **Jingcui Yu:** Funding acquisition (equal); investigation (equal); writing – review and editing (equal).

## FUNDING INFORMATION

This work was supported by the National Natural Science Foundation of China (Grant No. 30572092 and 31171227).

## CONFLICT OF INTEREST STATEMENT

The authors have no conflict of interest to declare.

## Supporting information


Figure S1.

Figure S2.

Figure S3.

Figure S4.

Figure S5.

Table S1.
Table S2.
Table S3.
Table S4.
Table S5.
Table S6.
Table S7.
Table S8.

## Data Availability

The data that support the findings of this study are available from the corresponding author upon reasonable request.
