# Supplementary figures and images for: Contribution of PGAP3 co‐amplified and co‐overexpressed with ERBB2 at 17q12 involved poor prognosis in gastric cancer
Source: J Cell Mol Med. 2023 Jun 29;27(16):2424–36. doi: 10.1111/jcmm.17828 (PMC10424286; doi:10.1111/jcmm.17828)

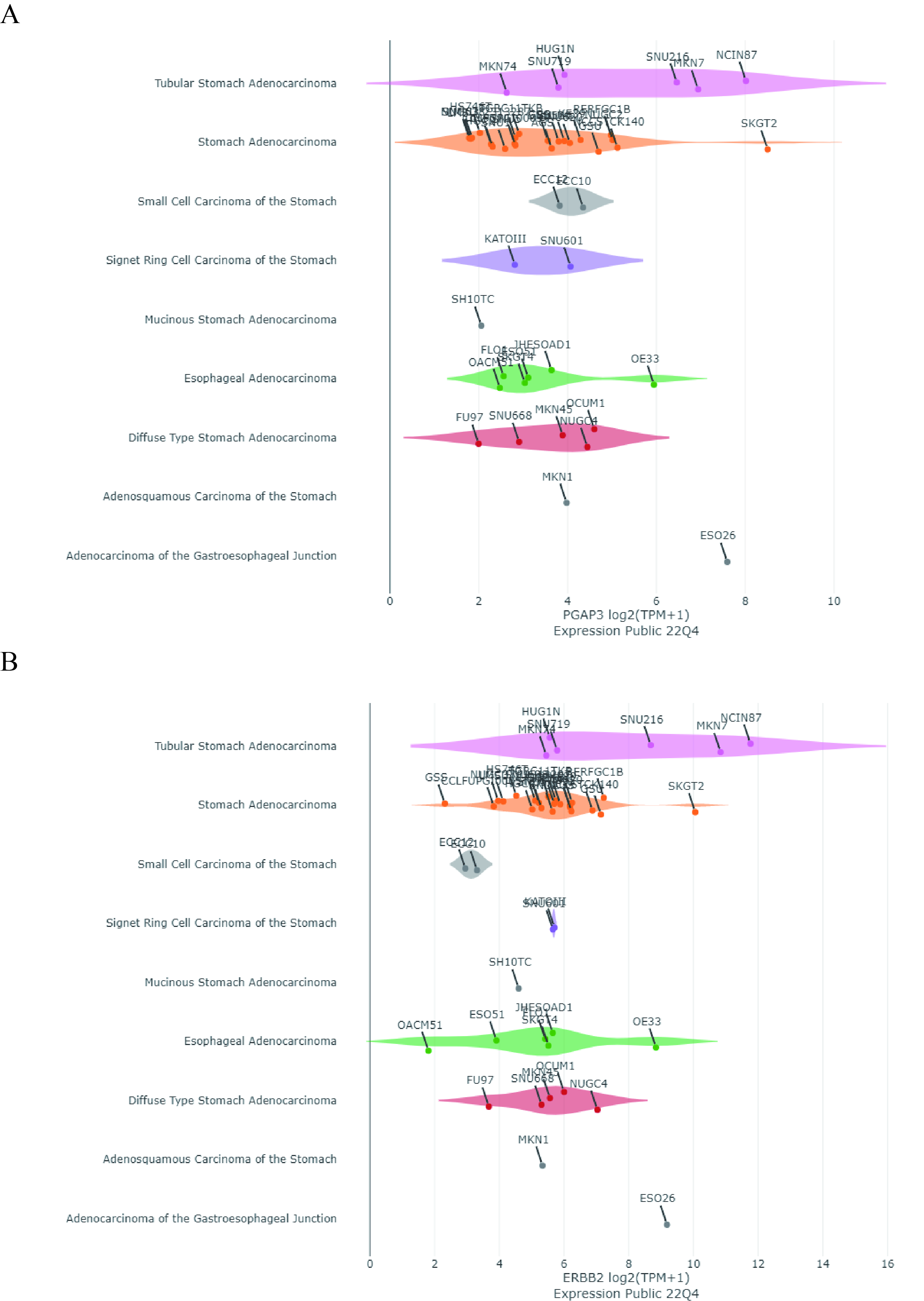

Supplement: Supplementary file 1 — Figure S1. [file JCMM-27-2424-s006.tif]

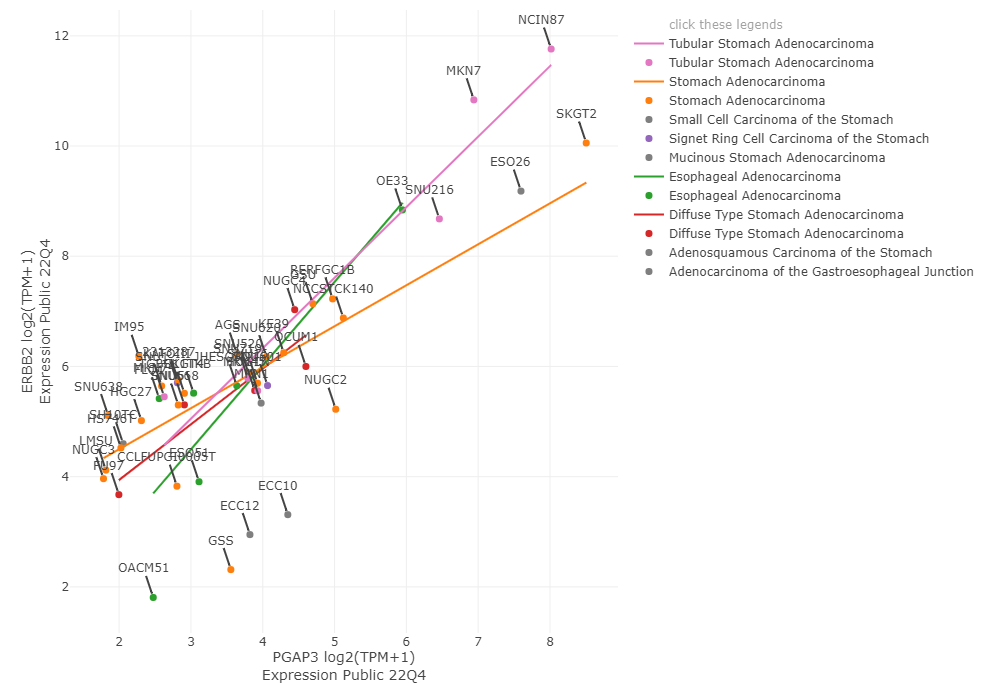

Supplement: Supplementary file 2 — Figure S2. [file JCMM-27-2424-s002.tif]

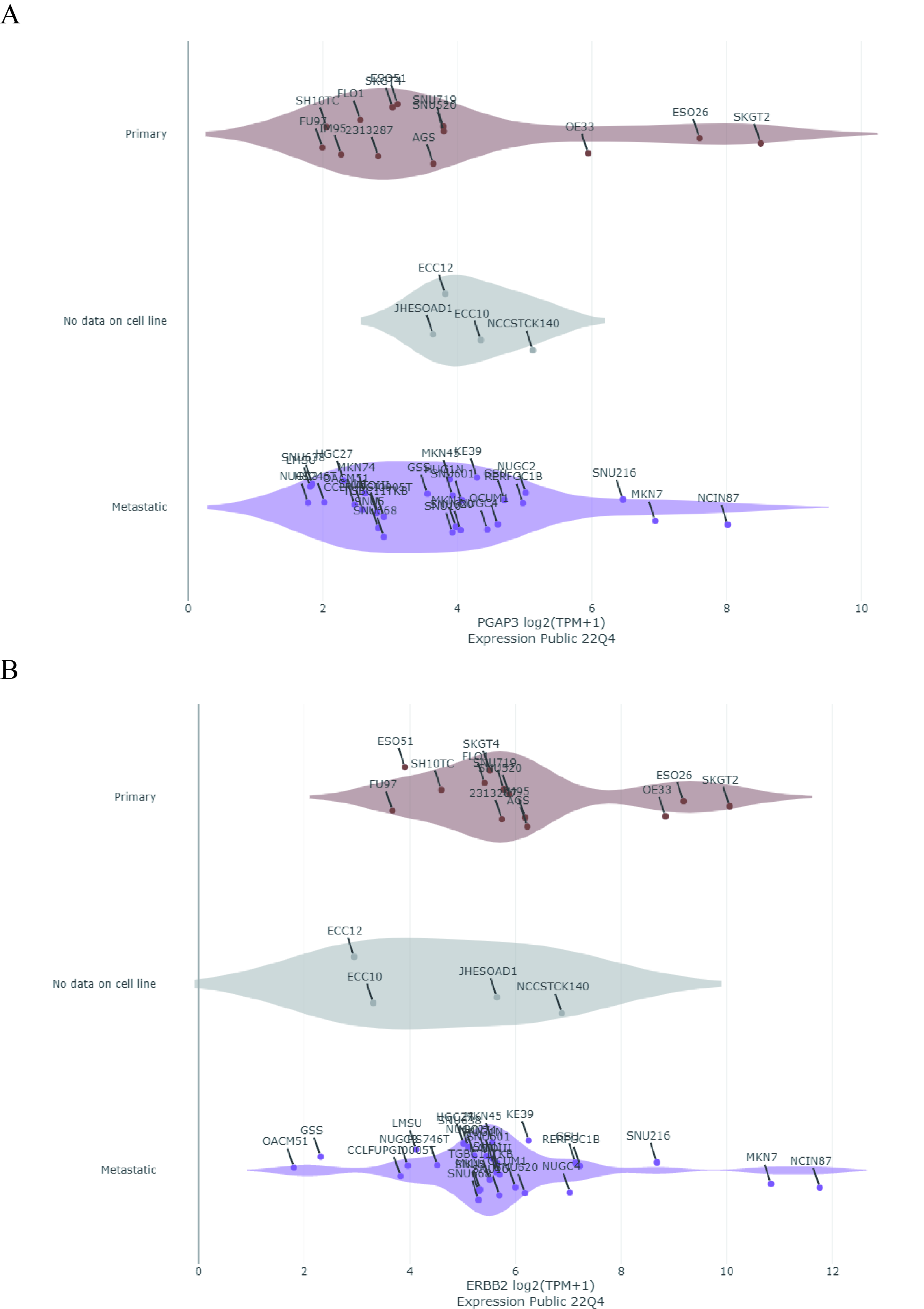

Supplement: Supplementary file 3 — Figure S3. [file JCMM-27-2424-s005.tif]

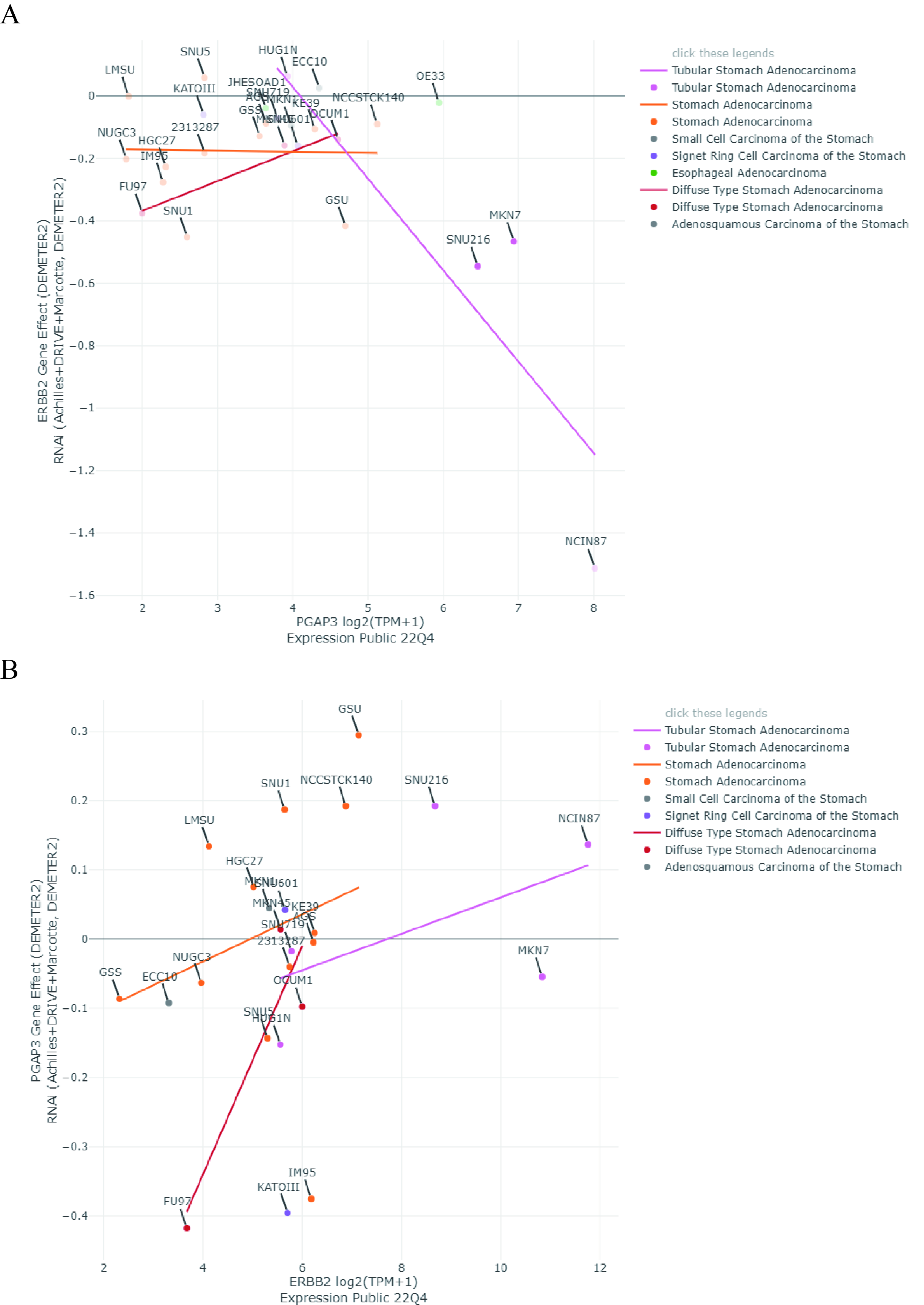

Supplement: Supplementary file 4 — Figure S4. [file JCMM-27-2424-s001.tif]

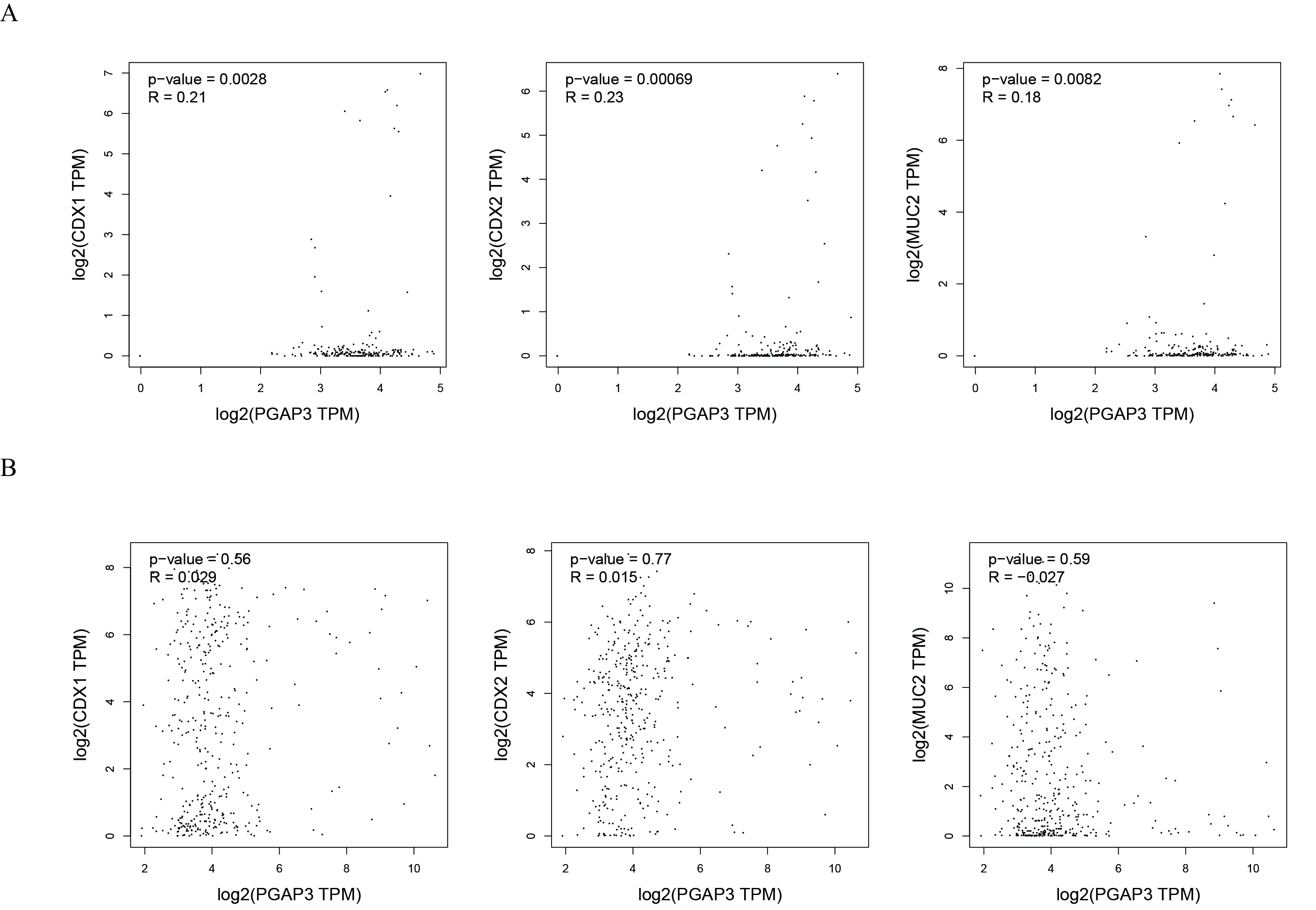

Supplement: Supplementary file 5 — Figure S5. [file JCMM-27-2424-s004.tif]
