# Supplementary material for: Contribution of PGAP3 co‐amplified and co‐overexpressed with ERBB2 at 17q12 involved poor prognosis in gastric cancer
Source: J Cell Mol Med. 2023 Jun 29;27(16):2424–36. doi: 10.1111/jcmm.17828 (PMC10424286; doi:10.1111/jcmm.17828)
Supplement: Supplementary file 6 — Table S1. Table S2. Table S3. Table S4. Table S5. Table S6. Table S7. Table S8. [file JCMM-27-2424-s003.docx]

**Supplementary table 1.** The sequences of the PCR primers

| **Gene** | **Forward** | | **Reverse** |
| --- | --- | --- | --- |
| **PGAP3 (DNA)** | | 5’-GAATGATTTGAGCCCCTGAA-3’ | 5’-ACCAGTCCAAAGTCCCTCCT-3’ |
| **PGAP3 (cDNA)** | | 5’-CACAAAGTGCCTCAGTTCCA-3’ | 5’-GAGGTCAGTGTCCCTGGTGT-3’ |
| **ERBB2 (DNA)** | | 5’-CCCAGCTCCTGTTTCTCTTG-3’ | 5’-GGCCTCTATTCCCACCTCTC-3’ |
| **ERBB2 (cDNA)** | | 5’-CGCTTTTGGCACAGTCTACA-3’ | 5’-TCCCGGACATGGTCTAAGAG-3’ |

**Supplementary table 2.** The sequences of the siRNAs

| **siRNA** | **Sense** | **Antisense** |
| --- | --- | --- |
| **Control** | 5’-UUCUCCGAACGUGUCACGUtt-3’ | 5’-ACGUGACACGUUCGGAGAAtt-3’ |
| **PGAP3-385** | 5’-CCUCGUUUCUCAAUGGCCUtt-3’ | 5’-AGGCCAUUGAGAAACGAGGtt-3’ |
| **PGAP3-189** | 5’-GCUCUGAAUCACUUCCGCUtt-3’ | 5’-AGCGGAAGUGAUUCAGAGCtt-3’ |
| **PGAP3-2661** | 5’-GGCAAGGGAUGUGCUUAAUtt-3’ | 5’-AUUAAGCACAUCCCUUGCCtt-3’ |
| **ERBB2-2712** | 5’-GGUGUAUGCAGAUUGCCAAtt-3’ | 5’-UUGGCAAUCUGCAUACACCtt-3’ |
| **ERBB2-2794** | 5’-GGUCAAGAGUCCCAACCAUtt-3’ | 5’-AUGGUUGGGACUCUUGACCtt-3’ |
| **ERBB2-2** | 5’-GUUGGAUGAUUGACUCUGAtt-3’ | 5’-UCAGAGUCAAUCAUCCAACtt-3’ |

**Supplementary table 3.** Correlation between PGAP3 expression and clinicopathological characteristics in GC

|  | variables | PGAP3 expression | | total | χ^2^ | *p* value |
| --- | --- | --- | --- | --- | --- | --- |
|  |  | low | high |  |  |  |
| Age (year) |  |  |  |  | 0.142 | \| 0.706 \| \| --- \| |
|  | ≤63 | 49 | 159 | 208 |  |  |
|  | >63 | 46 | 163 | 209 |  |  |
| Sex |  |  |  |  | 0.166 | 0.684 |
|  | Female | 31 | 98 | 129 |  |  |
|  | male | 64 | 224 | 288 |  |  |
| Grade |  |  |  |  | 0.052 | 0.82 |
|  | I/II | 21 | 75 | 96 |  |  |
|  | III/IV | 74 | 248 | 322 |  |  |
| T stage |  |  |  |  | 2.268 | 0.132 |
|  | T1/T2 | 20 | 47 | 67 |  |  |
|  | T3/T4 | 75 | 275 | 350 |  |  |
| N stage |  |  |  |  | 0.033 | 0.855 |
|  | N0 | 25 | 82 | 107 |  |  |
|  | N1/N2/N3 | 70 | 241 | 311 |  |  |
| M stage |  |  |  |  | 0.393 | 0.531 |
|  | M0 | 92 | 308 | 400 |  |  |
|  | M1 | 3 | 15 | 18 |  |  |
| TNM stage |  |  |  |  | 2.075 | 0.15 |
|  | I/II | 44 | 123 | 167 |  |  |
|  | III/IV | 51 | 200 | 251 |  |  |
| [Tumor](D:/Program%20Files%20(x86)/Youdao/Dict/8.9.6.0/resultui/html/index.html#/javascript:;) [size](D:/Program%20Files%20(x86)/Youdao/Dict/8.9.6.0/resultui/html/index.html#/javascript:;) |  |  |  |  | 1.721 | 0.19 |
|  | ≤5cm | 53 | 154 | 207 |  |  |
|  | >5cm | 42 | 166 | 208 |  |  |
| Lauren type |  |  |  |  | 8.656 | ^*^0.013 |
|  | Intestinal-type | 39 | 184 | 223 |  |  |
|  | Mixed-type | 18 | 53 | 71 |  |  |
|  | Diffuse-type | 38 | 84 | 122 |  |  |

Note: ^*^***p* < 0.05**, ^**^***p* < 0.01**, ^***^***p* < 0.001**

**Supplementary table 4.** Correlation between ERBB2 expression and clinicopathological characteristics in GC

|  | variables | ERBB2 expression | | total | χ^2^ | *p* value |
| --- | --- | --- | --- | --- | --- | --- |
|  |  | low | high |  |  |  |
| Age (year) |  |  |  |  | 1.880 | 0.17 |
|  | ≤63 | 135 | 73 | 208 |  |  |
|  | >63 | 122 | 87 | 209 |  |  |
| Sex |  |  |  |  | 2.008 | 0.157 |
|  | Female | 73 | 56 | 129 |  |  |
|  | male | 184 | 104 | 288 |  |  |
| Grade |  |  |  |  | 0.054 | 0.816 |
|  | I/II | 60 | 36 | 96 |  |  |
|  | III/IV | 197 | 125 | 322 |  |  |
| T stage |  |  |  |  | 5.9 | ^*^0.015 |
|  | T1/T2 | 50 | 17 | 67 |  |  |
|  | T3/T4 | 206 | 144 | 350 |  |  |
| N stage |  |  |  |  | 0.941 | 0.332 |
|  | N0 | 70 | 37 | 107 |  |  |
|  | N1/N2/N3 | 187 | 124 | 311 |  |  |
| M stage |  |  |  |  | 0.213 | 0.644 |
|  | M0 | 245 | 155 | 400 |  |  |
|  | M1 | 12 | 6 | 18 |  |  |
| TNM stage |  |  |  |  | 3.66 | 0.056 |
|  | I/II | 112 | 55 | 167 |  |  |
|  | III/IV | 145 | 106 | 251 |  |  |
| [Tumor](D:/Program%20Files%20(x86)/Youdao/Dict/8.9.6.0/resultui/html/index.html#/javascript:;) [size](D:/Program%20Files%20(x86)/Youdao/Dict/8.9.6.0/resultui/html/index.html#/javascript:;) |  |  |  |  | 3.934 | ^*^0.047 |
|  | ≤5cm | 138 | 69 | 207 |  |  |
|  | >5cm | 119 | 89 | 208 |  |  |
| Lauren type |  |  |  |  | 8.319 | ^*^0.016 |
|  | Intestinal-type | 137 | 86 | 223 |  |  |
|  | Mixed-type | 34 | 37 | 71 |  |  |
|  | Diffuse-type | 84 | 38 | 122 |  |  |

Note: ^*^***p* < 0.05**, ^**^***p* < 0.01**, ^***^***p* < 0.001**

**Supplementary table 5.** Univariate and multivariate analyses of the factors correlated with Overall survival of [gastric](javascript:;) cancer patients

| variables | Univariate analysis | | | | |  | Multivariate analysis | | | |
| --- | --- | --- | --- | --- | --- | --- | --- | --- | --- | --- |
|  | *p* value | | HR | 95%CI | |  | *p* value | HR | 95%CI | |
|  |  |  | | inferior limit | [upper](javascript:;) [limit](javascript:;) |  |  |  | inferior limit | [upper](javascript:;) [limit](javascript:;) |
| PGAP3  （low vs high） | ^*^0.013 | 1.539 | | 1.096 | 2.161 |  | ^*^0.042 | 1.425 | 1.012 | 2.007 |
| Age  （≤63 vs  >63） | 0.194 | 1.19 | | 0.915 | 1.549 |  |  |  |  |  |
| Sex  （Female vs  male） | 0.341 | 0.874 | | 0.663 | 1.153 |  |  |  |  |  |
| [Tumor](D:/Program%20Files%20(x86)/Youdao/Dict/8.9.4.0/resultui/html/index.html#/javascript:;) [size](D:/Program%20Files%20(x86)/Youdao/Dict/8.9.4.0/resultui/html/index.html#/javascript:;)  （≤5cm vs  >5cm） | <0.001 | 1.813 | | 1.386 | 2.373 |  | ^*^0.03 | 1.359 | 1.03 | 1.792 |
| Grade stage  （I/II vs  III/IV） | <0.001 | 1.954 | | 1.367 | 2.793 |  | 0.056 | 1.426 | 0.991 | 2.05 |
| T stage  （T1/T2 vs  T3/T4） | <0.001 | 3.458 | | 2.045 | 5.845 |  | ^*^0.014 | 2.027 | 1.151 | 3.569 |
| N stage  (N0 vs  N1/N2/N3) | <0.001 | 4.903 | | 3.149 | 7.633 |  | <0.001 | 4.02 | 2.535 | 6.376 |
| M stage  (M0 vs  M1) | <0.001 | 4.063 | | 2.127 | 7.76 |  | ^**^0.004 | 2.636 | 1.372 | 5.063 |

Note: ^*^***p* < 0.05**, ^**^***p* < 0.01**, ^***^***p* < 0.001**

**Supplementary table 6.** Univariate and multivariate analyses of the factors correlated with Overall survival of [gastric](javascript:;) cancer patients

| variables | Univariate analysis | | | |  | Multivariate analysis | | | |
| --- | --- | --- | --- | --- | --- | --- | --- | --- | --- |
|  | *p* value | HR | 95%CI | |  | *p* value | HR | 95%CI | |
|  |  |  | inferior limit | [upper](javascript:;) [limit](javascript:;) |  |  |  | inferior limit | [upper](javascript:;) [limit](javascript:;) |
| PGAP3  （low vs high） | ^*^0.013 | 1.539 | 1.096 | 2.161 |  | ^*^0.042 | 1.425 | 1.012 | 2.007 |
| Age  （≤63 vs  >63） | 0.194 | 1.19 | 0.915 | 1.549 |  |  |  |  |  |
| Sex  （Female vs  male） | 0.341 | 0.874 | 0.663 | 1.153 |  |  |  |  |  |
| [Tumor](D:/Program%20Files%20(x86)/Youdao/Dict/8.9.4.0/resultui/html/index.html#/javascript:;) [size](D:/Program%20Files%20(x86)/Youdao/Dict/8.9.4.0/resultui/html/index.html#/javascript:;)  （≤5cm vs  >5cm） | <0.001 | 1.813 | 1.386 | 2.373 |  | ^*^0.03 | 1.359 | 1.03 | 1.792 |
| Grade stage  （I/II vs  III/IV） | <0.001 | 1.954 | 1.367 | 2.793 |  | 0.056 | 1.426 | 0.991 | 2.05 |
| T stage  （T1/T2 vs  T3/T4） | <0.001 | 3.458 | 2.045 | 5.845 |  | ^*^0.014 | 2.027 | 1.151 | 3.569 |
| N stage  (N0 vs  N1/N2/N3) | <0.001 | 4.903 | 3.149 | 7.633 |  | <0.001 | 4.02 | 2.535 | 6.376 |
| M stage  (M0 vs  M1) | <0.001 | 4.063 | 2.127 | 7.76 |  | ^**^0.004 | 2.636 | 1.372 | 5.063 |

Note: ^*^***p* < 0.05**, ^**^***p* < 0.01**, ^***^***p* < 0.001**

**Supplementary table 7.** Survival proportions of PGAP3 expression in GC

| Group | Number | Death | Survival | [survival](javascript:;) [rate](javascript:;) |
| --- | --- | --- | --- | --- |
| Low | 74 | 41 | 33 | 44.60% |
| High | 269 | 182 | 87 | 32.30% |
| Total Number | 343 | 223 | 120 | 35.00% |

**Supplementary table 8.** Survival proportions of ERBB2 expression in GC

| Group | Number | Death | Survival | [survival](javascript:;) [rate](javascript:;) |
| --- | --- | --- | --- | --- |
| Low | 197 | 117 | 80 | 40.60% |
| High | 146 | 106 | 40 | 27.40% |
| Total Number | 343 | 223 | 120 | 35.00% |
